# A Multi-Task Convolutional Neural Network for Lesion Region Segmentation and Classification of Non-Small Cell Lung Carcinoma

**DOI:** 10.3390/diagnostics12081849

**Published:** 2022-07-31

**Authors:** Zhao Wang, Yuxin Xu, Linbo Tian, Qingjin Chi, Fengrong Zhao, Rongqi Xu, Guilei Jin, Yansong Liu, Junhui Zhen, Sasa Zhang

**Affiliations:** 1 Key Laboratory of Education Ministry for Laser and Infrared System Integration Technology, Shandong University, 72 Binhai Road, Qingdao 266237, China; 202034042@mail.sdu.edu.cn (Z.W.); 201920409@mail.sdu.edu.cn (L.T.); 17852727001@163.com (R.X.); 2 Shandong Provincial Key Laboratory of Laser Technology and Application, Shandong University, 72 Binhai Road, Qingdao 266237, China; 3 Department of Pathology, Qilu Hospital, Shandong University, Jinan 250012, China; yuxinxu@mail.sdu.edu.cn; 4 The Key Laboratory of Experimental Teratology, Ministry of Education and Department of Pathology, School of Basic Medical Sciences, Shandong University, Jinan 250012, China; 5 School of Information Science and Engineering, Shandong University, 72 Binhai Road, Qingdao 266237, China; 202032770@mail.sdu.edu.cn (Q.C.); 202132758@mail.sdu.edu.cn (F.Z.); 202000120143@mail.sdu.edu.cn (G.J.); 6 Department of Breast Disease, Shandong Cancer Hospital and Institute, Shandong First Medical University (Shandong Academy of Medical Sciences), 440 Jiyan Road, Jinan 250012, China; liuyansong@hotmail.com

**Keywords:** histopathological images, lung cancer, medical images, multiple tasks, segmentation, classification, deep learning, convolutional neural network

## Abstract

Targeted therapy is an effective treatment for non-small cell lung cancer. Before treatment, pathologists need to confirm tumor morphology and type, which is time-consuming and highly repetitive. In this study, we propose a multi-task deep learning model based on a convolutional neural network for joint cancer lesion region segmentation and histological subtype classification, using magnified pathological tissue images. Firstly, we constructed a shared feature extraction channel to extract abstract information of visual space for joint segmentation and classification learning. Then, the weighted losses of segmentation and classification tasks were tuned to balance the computing bias of the multi-task model. We evaluated our model on a private in-house dataset of pathological tissue images collected from Qilu Hospital of Shandong University. The proposed approach achieved Dice similarity coefficients of 93.5% and 89.0% for segmenting squamous cell carcinoma (SCC) and adenocarcinoma (AD) specimens, respectively. In addition, the proposed method achieved an accuracy of 97.8% in classifying SCC vs. normal tissue and an accuracy of 100% in classifying AD vs. normal tissue. The experimental results demonstrated that our method outperforms other state-of-the-art methods and shows promising performance for both lesion region segmentation and subtype classification.

## 1. Introduction and Literature Review

Cancer is a disease with one of the highest death rates. Lung cancer is the leading cause of cancer death in the world, and its five-year survival rate is very low. According to the World Health Organization, about 85% of lung cancer cases are patients with non-small cell lung cancer (NSCLC) [1]. The most common histological subtypes of NSCLC are adenocarcinoma and squamous cell carcinoma. Recent years have witnessed great advances in the molecular therapeutic methods based on targeted therapies for NSCLC, prolonging not only progression-free survival but also overall survival. It is necessary for pathologists to identify NSCLC histopathological type by examining pathological tissue slices. However, the manual inspection process of tissue slices is very time-consuming and highly depends on the experience of pathologists. In order to improve the histopathological evaluation, it is possible to use a computer-aided diagnosis (CAD) system [2] that identifies cancer lesions and classifies them according to their histopathological type.

Two important tasks should be performed by a deep learning model for tumor images: cancer lesion segmentation and tumor classification. The lesion segmentation task aims to detect tumor location and boundaries, and the tumor classification task aims to identify tumor histological subtypes. In previous studies, many traditional machine learning methods were presented, such as the combination of a probabilistic neural network and support vector machines (PNN-SVM) [3,4], the Bayesian classifier [5,6] and the neural-like structure of successive geometric transformations model (SGTM) [7,8,9,10] for tumor classification and Gibbs random field [11], fuzzy C-means [12] and Wavelet Analysis [13] for segmentation. These methods highly rely on hand-crafted feature engineering and are unable to learn deep representations from visual levels. In recent years, deep learning has contributed to significant developments in the field of medical image processing [14]. Most deep learning frameworks for medical image processing are based on convolutional neural networks (CNNs), and the formats of medical data are 2-D or 3-D images, in general. A CNN is an effective multi-layer artificial neural network for the extraction of image characteristics, popularly used for classification and object detection [15,16,17,18]. Classical frameworks of the convolution network, such as DenseNet [19], ResNet [20], Inception V3 [21] and VGG16, are very useful for classifying images [22]. Using the above frameworks, Alom et al. [23] performed an elementary classification of pathological tissues images of benign and malignant breast cancer. Coudray et al. [24] and Wang et al. [25] carried out a complex classification of histological subtypes. Nevertheless, whole-slide images used as models are too small and difficult to examine by pathologists, thus the methods offer poor help for physician-assisted diagnosis. Boumaraf et al. [26] and Ukwuoma et al. [27] classified images of pathological breast cancer tissues at various magnifications by visualization, trying to apply the operating principle of the models [28,29], as simple classification processes cannot help pathologists analyze medical images. However, the efficacy of image visualization is not convincing. Segmenting cancer lesions precisely helps pathologists quickly identify key areas of concern. In addition, pathologists can understand the reasons of model discrimination errors. Classical frameworks such as U-Net [30,31], Mask R-CNN [32], FCN [33,34] play an important role in medical image segmentation. The U-Net model itself is proposed for the segmentation of medical images, but it focuses on the cellular level. Since feature information in pathological images is too dense, Li et al. [35] utilized multi-level feature fusion to segment nuclei in digital histopathological images, and Wang et al. [36] randomly selected millions of patches of pathological images to obtain a dataset, while the model should be retrained with hand-picked images to avoid a non-uniform distribution of images with blank areas. Kumar et al. [37] trained the model using a thermal map mask instead of a binary mask as ground truth to directly locate cancer lesions on histopathological images. Cell segmentation and the prediction of cancer recurrence probability are achieved using two models which are complex. Liu et al. [38] and Zhang et al. [39] constructed simple models for lesion region segmentation and disease classification, but they are effective only for 3D images.

We established a model for simultaneously segmenting and classifying pulmonary epithelial tumors on the basis of 2D pathological tissues images of lung cancer. Different types of tumor cells have different histological features. Thus, tumor segmentation and histological subtype classification are well related in medical diagnosis. The proposed model consists of a shared feature-extracting channel to obtain multi-scale feature maps, which improves both automatic segmentation and classification. Since they share the same extraction channel, the segmentation and classification tasks affect the gradient descent backpropagation of parameters. We balanced the two tasks by adjusting the proportion of the loss weights of the two tasks. Experiments on our self-collected pathological images dataset demonstrated that our method achieved a promising performance and outperformed other state-of-the-art multi-task methods. In summary, this work has three main contributions:

- We propose a novel end-to-end multi-task convolutional neural network (MCN) for lung cancer lesion region segmentation and tumor histological subtype classification, achieved by sharing the same extracted spatial information.
- Our model solved the complex problem of multi-class segmentation and classification, obtaining balanced loss weight ratios.
- Our model recognized and segmented cancer lesions more precisely than manually annotation, which is boundary-blurry, shape-irregular and location-random.

The remainder of this article is organized as follows. Section 2 illustrates the materials and the proposed method. Section 3 discusses evaluation indexes and the experimental results with different weight of losses and compares our approach with other methods. Section 4 concludes this work and presents prospects for the future.

## 2. Materials and Methods

**Figure 1:**
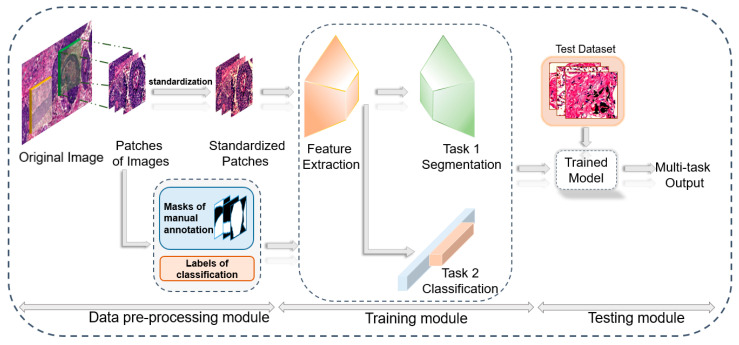
Structure of our research. A data pre-processing module was developed to annotate, crop and standardize original images into image patches and corresponding ground truth. With the training module and testing module, the multi-task model was trained and tested.

Our research included the development of a data pre-processing module, a multi-task model training module and a testing module, as shown in Figure 1. Firstly, 10x histopathologic slices were scanned on a computer and annotated by pathologists, and then corresponding masks for the segmentation task were generated. To reduce the computational complexity, we cropped the original pathological sections and corresponding masks into image patches and mask patches. Before computing, input images were standardized. Secondly, we input standardized image patches and corresponding ground truth including mask patches and classification labels into the multi-task model for training. Ultimately, we used the test dataset that was not used in model training to evaluate the segmentation and classification performance of the model. Details will be explained subsequently.

### 2.1. Data Source

As shown in Table 1, the image and case information data [40] were obtained from cases demonstrably diagnosed with NSCLC in Qilu Hospital of Shandong University in 2021–2022, and the tissue sections of typical NSCLC images were scanned; all of them were surgically resected specimens. We random selected 36 patients, including 15 with SCC, 11 with AD and 10 normal controls (NC), using H&E staining image data of lung epithelial tumor cases. Basic clinical characteristic included histopathological type, patient age, cancer staging, representing the clinical Tumor Node Metastasis (TNM) stage [41], and tumor volume, representing the largest tumor region seen in general. The surgically resected specimens were paraffin-embedded after general sampling, tissue fixation, dehydration until transparency, wax immersion and embedding, and then H&E tissue sections were obtained after sectioning and staining. A Roche digital scanner was used to scan the screened tissue sections, and a total of 312 original images of 10× pathological tissue sections were obtained from the H&E scan sections of different cases (open dataset: https://github.com/Joyw7070/LungImageAnalysis (accessed on 27 June 2022)). The original images were very large and thus they would lead to computational parameters of millions of magnitude in the process of model training. Thus, we randomly cropped the original images into 480 × 480-pixel image patches and standardized the image patches to accelerate the convergence of the model in training.

**Figure 2:**
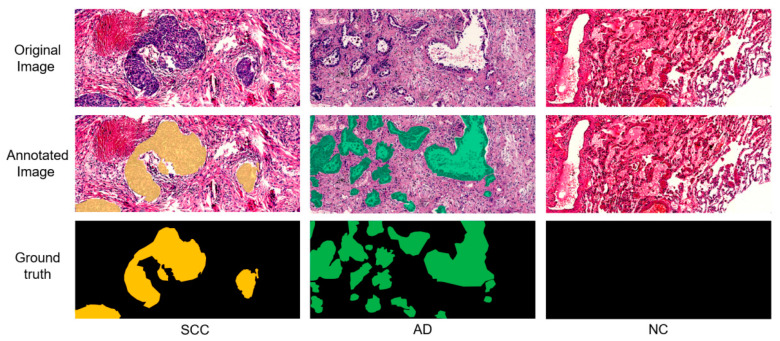
The first row shows examples of the original images scanned from histopathologic slices. The second row shows the annotated images where pathologists highlighted the lesion regions. The third row shows binary masks for the segmentation task.

Figure 2 shows pathological H&E original images, expert-annotated pathological images and masks of SCC samples, AD samples and NC samples. As can be seen from the original images, the lesion regions were composed of malignant epithelial cells and reactive stroma. In the original images, NSCLC showed different histological characteristics. In fact, SCC appeared mostly distributed in nests, while AD mostly formed glandular lumen structure and infiltrated the hyperplastic fibrous stroma. The invasive growth of cancer cells can occur in the form of nest sheets, adenoids, sieves, etc., or can involve infiltrating scattered single cells, so the pathological regions in the pathological sections mostly showed an irregular shape.

The annotation work was carried out by two pathologists. After the boundaries of tumor lesion regions were marked, masks were generated, where the pixel value of 1 represented SCC lesions (yellow areas), the pixel value of 2 represented AD (green area), and the pixel value of 0 represented the background (black area). These masks were used for the segmentation.

In this study, we randomly divided the whole dataset into training set (90%) and a test set (10%). Subsequently, a 5-fold cross-validation method was employed to obtain the optimal combination of hyper-parameters of the neural network, in which the training set was evenly divided into 5 subsets; 4 data subsets were used as training data to update the model weight parameters, and 1 subset as validation data to adjust the hyper-parameters of the model and carry out the experiment. The test set was not used for training or validation but to evaluate the final generalization ability of the model.

### 2.2. Multi-Task CNN for Cancer Lesion Segmentation and Histological Subtype Classification

**Figure 3:**
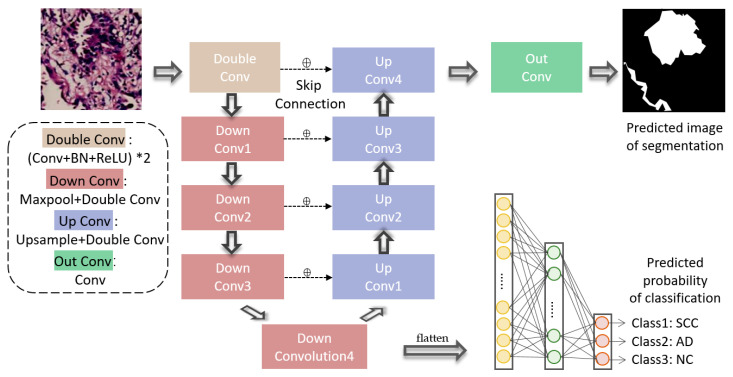
Structure of the MCN. “Double Conv” denotes the two operations of convolution, in which the kernel size was 3 × 3, the stride was 1, and the padding was 1, batch-normalization and ReLU. “Down Conv” denotes max-pooling and the operation of “Double conv”. “Up Conv” denotes up-sampling, in which the kernel size was 2 × 2, and the operation of “Double conv”. “Out Conv” denotes an operation of convolution in which the kernel size was 1 × 1, the stride was 1, and the padding was 0. The “flatten” operation denotes the adaptive average pooling and was followed by two fully connected layers. The “⊕” operation concatenates feature maps in left side and the right side as input to next “Up Conv” module.

Different from other methods that segment and classify lung cancer pathological images separately, we propose a dual-branch multi-task convolution model based on a single feature extraction channel, as shown in Figure 3. The input of the model was a 480 × 480-pixel image patch, and the output was a 480 × 480-pixel mask which marked the lesion regions and allowed the prediction of tumor subtype. This model can perform two tasks including lesion region segmentation and tumor subtype classification and achieve a synchronous gradient descent. After the extraction of features through the shared channel, the classification branch inputs the feature maps into fully connected layers, allowing tumor type classification. On the other hand, the segmentation branch gradually restores the feature map to the original input size by the up-sampling and the jumping connection methods and adds a convolution layer to classify each pixel, thus completing the semantic segmentation task of each pixel in the input image.

The ‘contractive channel’ was established to perform down-sampling feature extraction from pathological images patches including one double convolution module (Double Conv) and four down convolution modules (Down Conv1-4). In this process, the image feature map was shrunk step by step. The input of the model was a 480 × 480 three-channel RGB image patch. The Double Conv module implemented two 2D convolutions, 2D batch-normalization and ReLU operations on the input image, where the convolution kernel size was 3 × 3, the stride was 1, and the padding was 1. Each Down Conv module consisted of a 2D max-pooling and a Double Conv (the same operation method as the Double Conv module), where the kernel size of the max-pooling layer was 2 × 2, and the stride was 2. Max-pooling reduced the dimension of the output from the previous layer to reduce the scale of the parameters while retaining the main features. The ‘contractive channel’ continuously reduced the resolution to obtain image information from different scales. Image information was gradually transformed from the line, texture, color, etc., in underlying information about the contour and more abstract information in high-level information.

After the Down Conv4, we believe that the neural network obtained enough multi-dimensional abstract feature information. Then, during the task of classification, we performed global average pooling on the high-dimensional feature map through adaptive average pooling and a nonlinear calculation through two full connected layers after spreading, to calculate the probability of the specific tumor subtypes.

However, the semantic segmentation task consists in the classification of tumor categories at each pixel position in an image (the tissues where the pixels are located are judged as SCC, AD or NC). Therefore, for the segmentation task, the contraction of feature maps does not provide enough information; therefore, we used a ‘symmetric’ extended channel with the contracting channel. The extended channel restored the image resolution of the output layer to the same resolution as that of the input image by using four up-sampling convolution modules (Up Conv1-4) and one output convolution module (Out Conv). The Up Conv module contained an up-sampling convolution and a double convolution operation (Double Conv). Each up-sampling convolution operation adopted bilinear interpolation.

In the restoring process, the feature map would be distorted when transforming from low resolution to high resolution, so it would lose details in the up-sampling process. To solve this problem, the neural network connected the feature map of the left symmetric channel with the corresponding up-sampling result through the skip connection method, as the input of the next module. In other words, skip connection added detailed information in each pixel of the judgment target and allowed the model to achieve more accurate segmentation results. Finally, the Out Conv module applied a convolution calculation, where the convolution kernel size was 1 × 1, the stride was 1, and the padding was 0. The number of output image channels was 3, that is, the input of a three-layer mask. The same position on each channel represented the probability that that point in the original image was SCC, AD or NC tissue.

As shown in Figure 3, the MCN outputted the predicted mask and tumor subtype probability. For a segmented sample, the objective of optimization is to minimize the cross entropy loss function, and the calculation formula is as follows:

(1) $${Loss}_{\mathrm{seg}}=-\log\left(\frac{\exp\left(z\left[c\right]\right)}{\sum_{j=0}^{c-1}\exp\left(z\left[j\right]\right)}\right)\;=-z\left[c\right]+\log\left(\overset{c-1}{\underset{j=0}{\sum }}\exp\left(z\left[j\right]\right)\right)$$

where z = [z_0_, z_1_, z_2_] represents the output of the segmentation mask for three classes, including SCC, AD and background, and c is the ground truth of the sample. For the classified sample, the loss function is as follows:

(2) $${Loss}_{\mathrm{cls}}=-\hat{y}\cdot \mathrm{logy}$$

where $\hat{y}$ is the sample label of the subtype, and y is the probability of properly classifying the subtype of the sample. The loss of MCN is the sum of weighted segmentation and classification losses:

(3) $${Loss=\Gamma }_{1}\cdot {Loss}_{\mathrm{seg}}{+\Gamma }_{2}\cdot Loss_{\mathrm{cls}}$$

where Γ_1_ and Γ_2_ are the weight value of segmentation and classification loss, respectively. In the training process of the multi-task model, the weights of the loss values affect the performance of the corresponding tasks. We will discuss the results in detail in Section 3.2. All experimental hyper-parameters (including comparable methods) in this work remained consistent during training, i.e., adaptive learning rate (method: torch. LambdaLR, original rate = 0.01) and batch size of 4. The optimization of the MCN was performed with the SGD method (momentum = 0.9, weight decay = 0.0001).

## 3. Results

### 3.1. Setting the Valuation Indexes

The performance of both cancer lesion region segmentation and histological subtype classification is evaluated by several indexes. To evaluate the performance of segmentation, we used the Dice similarity coefficient (DSC), sensitivity (SEN), precision (PRE) and Intersection over Union (IoU). DSC and IoU indicate the similarity of predicted segmenting masks, while ground truth and the DSC pay more attention to pixels correctly predicted as lesion. SEN represents the proportion of a lesion area which is correctly predicted in ground truth, and PRE represents the proportion of a lesion area which is correctly predicted in the predicted mask. We computed the evaluation indexes of SCC, AD and NC, according to the following formulas:

(4) $${\mathrm{DSC}}_{\;}{=\Sigma }_{s=1}^{m}\frac{{2\mathrm{TP}}_{i}{}^{\left(s\right)}}{{2\mathrm{TP}}_{i}{}^{\left(s\right)}{+\mathrm{FP}}_{i}{}^{\left(s\right)}{+\mathrm{FN}}_{i}{}^{\left(s\right)}}$$

(5) $${\mathrm{SEN}\;}_{\;}{=\Sigma }_{s=1}^{m}\frac{{\mathrm{TP}}_{i}{}^{\left(s\right)}}{{\mathrm{TP}}_{i}{}^{\left(s\right)}{+\mathrm{FN}}_{i}{}^{\left(s\right)}}$$

(6) $${\mathrm{PRE}\;}_{\;}{=\Sigma }_{s=1}^{m}\frac{{\mathrm{TP}}_{i}{}^{\left(s\right)}}{{\mathrm{TP}}_{i}{}^{\left(s\right)}{+\mathrm{FP}}_{i}{}^{\left(s\right)}}$$

(7) $${\mathrm{IoU}}_{\;}=\frac{{\mathrm{DSC}}_{\;}}{2\;-{\;\mathrm{DSC}}_{\;}}$$

where m is the number of samples; s is the sth sample; ${\mathrm{TP}}_{i}$ denotes the true positive of Class i, i.e., the predicted pixels of Class i inside the positive regions of ground-truth; ${\mathrm{FP}}_{i}$ denotes the false positive of Class i, i.e., the predicted pixels of Class i outside the positive regions of ground-truth; $FN_{i}$ denotes the false negative of Class i, i.e., the pixels which are predicted to correspond to other two Classes inside the positive regions of ground-truth. In addition, we calculated the average of the evaluation indexes of the three classes.

For the classification task, the goal was to distinguish SCC from NC and AD from NC; this involved three evaluation indexes: classification accuracy (ACC), sensitivity (SEN), specificity (SPE). ACC is the proportion of correctly classified subjects among all subjects. SEN is the proportion of correctly classified SCC/AD patients. SPE is the proportion of correctly classified NC subjects. The formulas are as follows:

(8) $${\mathrm{ACC}}_{\;}=\frac{{\mathrm{TP}}_{c}{}_{\;}{+\mathrm{TN}}_{c}}{{\mathrm{TP}}_{c}{+\mathrm{TN}}_{c}{+\mathrm{FN}}_{c}{+\mathrm{FP}}_{c}}$$

(9) $${\mathrm{SEN}}_{\;}=\frac{{\mathrm{TP}}_{c}{}_{\;}}{{\mathrm{TP}}_{c}{+\mathrm{FN}}_{c}}$$

(10) $$\mathrm{SPE}=\frac{{\mathrm{TN}}_{c}{}_{\;}}{{\mathrm{TN}}_{c}{+\mathrm{FP}}_{c}}$$

where ${\mathrm{TP}}_{c}$ denotes the number of true positive samples; ${\mathrm{TN}}_{c}$ denotes the number of true negative samples; ${\mathrm{FN}}_{c}$ denotes the number of false positive samples; ${\mathrm{FP}}_{c}$ denotes the number of false negative samples.

### 3.2. Compromising on the Weight of Losses

In our experiments, we assigned different weights to the two tasks’ losses to determine the best weight ratio of model learning. Γ_1_ and Γ_2_ represent the weights of $Loss_{seg}$ and $Loss_{cls}$. Two parameters influence the calculation in training. We determined the task whose loss had the larger weight. Table 2 shows the comparison of the segmentation and classification results by compromising on different Γ_1_ and Γ_2_ for single and multi-task learning. Referring to the classification task, compared to other three values of Γ_1_ and Γ_2_, the model performed best when Γ_1_ was equal to Γ_2_, achieving 100% accuracy in AD identification. For single tasks, better performance was achieved than for multi-task learning with equal Γ_1_ and Γ_2_ in SCC classification, obtaining ACC, SEN and SPE of 97.8%, 95.2% and 100%, respectively, worse performance was observed in AD classification. As for the segmentation task, firstly, according to the means, multi-task learning with equal Γ_1_ and Γ_2_ performed best, achieving DSC of 92.3%, SEN of 92.2%, PRE of 91.9% and IoU of 85.2%. Secondly, when the multi-task was carried out using different unbalanced values of Γ, we observed that model learning with higher Γ_1_ performed better than model learning with the lower Γ_1_. Specifically, the single segmentation task learning achieved the best performance for SCC, with DSC, SEN, PRE, IoU of 95.6%, 94.2%, 97.8%, 92.3%, respectively.

### 3.3. Comparison with Other Methods

We compared our proposed method to two other existing methods for medical image analysis, i.e., MDCN [38] and MGMLN [39]. MDCN was proposed to segment the hippocampus and classify Alzheimer’s disease using a deep-learning model. The model was constructed to obtain a 3D DenseNet to learn features of 3D mild cognitive impairment patches. We rebuilt the model to adapt it to 2D images without changing its structure. MGMLN was proposed as a multi-task deep learning model for automatic gastric tumor segmentation and lymph node classification applied on CT scans. Since the model was constructed for 2D images, we directly trained and tested it on our dataset. The evaluation results shown in Table 3 contain the segmentation evaluation and the classification evaluation. Our method outperformed MDCN and MGMLN in both tasks.

#### 3.3.1. Segmentation Evaluation

**Figure 4:**
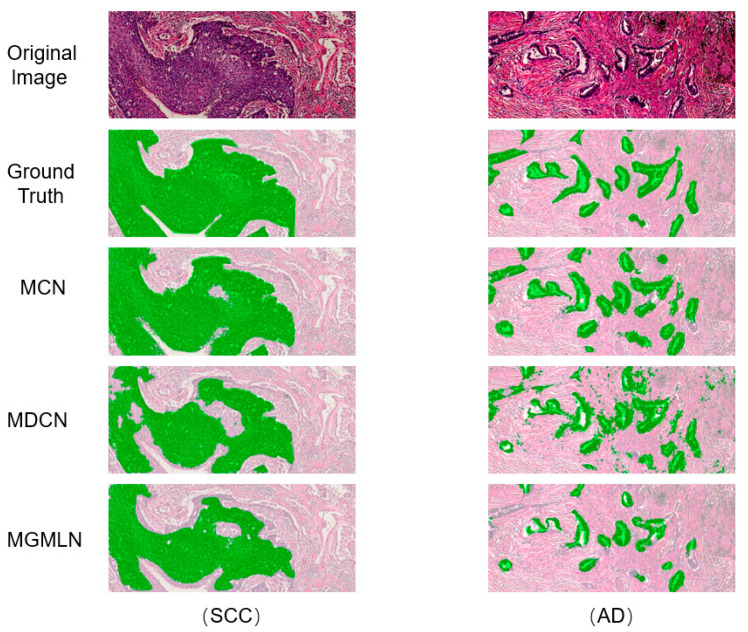
Comparison of the segmentation results for SCC and AD obtained with our method (MCN) and other two methods (MDCN and MGMLN).

Figure 4 shows the comparison of the segmentation results using different methods for two specimens of SCC and AD from the test data. From top to bottom, the Figure shows are original images, ground truth, segmented results of our method, MDCN and MGMLN. It is difficult to intuitively distinguish the lesion area based on the contrast between adjacent cells and stroma in the whole image of pathological tissue, considering image color and brightness. The lesion region segmentation results achieved by our method showed smoother edges and more accurate shapes. The segmentation result of SCC obtained with MDCN showed under-fitting, which means inadequate segmentation of the lesion area, whereas the segmentation result of AD showed over-fitting, which means misdiagnosing non-diseased regions as diseased regions. With MGMLN, the segmentation results of both SCC and AD showed under-fitting. In addition, the segmentation performance reported in Table 3 indicates that our method outperformed other methods in DSC, SEN, PRE, IoU.

#### 3.3.2. Classification Evaluation

**Figure 5:**
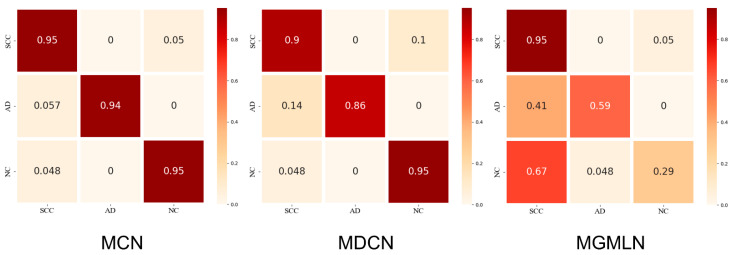
Confusion matrixes of the classification results of MCN, MDCN, MGMLN. In each matrix, rows denote the histological subtype labels, and columns denote the predicted histological subtypes.

Figure 5 shows the accuracy of the classification results by the three methods. The more intense the red on the main diagonal of the confusion matrix, the better the prediction effect. On the contrary, the more distributed the red is in the graph, the worse the prediction effect. In the confusion matrix of MGMLN, some red squares are present in the lower left portion, indicating poor specificity, which means that images of normal and AD tissue are interpreted as indicating SCC; while in the confusion matrix of MDCN, some light red squares are present on both sides of the main diagonal of the graph, indicating that the method’s prediction ability is relatively good, though with a little insufficient SEN and SPE. In the confusion matrix of our method, it can be seen that red color is mostly concentrated on the main diagonal, and the deviation on both sides is under 6 × 10^−2^. The classification results are shown in Table 3 according to the three evaluation indexes. Our proposed method appeared superior to the examined other methods in general, and the accuracy of the classification results of SCC and AD was 100% and 95.1%, respectively.

## 4. Conclusions

In this paper, we proposed a competitive model based on a multi-task convolutional neural network for jointly determining cancer lesion area segmentation and histological subtype classification. By compromising on the weight of losses, the highest classification accuracies using our method were between 92.7% and 97.8% for classifying SCC and between 90.2% and 100.0% for classifying AD, while the obtained classification results using other methods ranged from 64.1% to 100.0%. The DSCs of segmenting lesions completed by our method were between 89.0% and 94.5%, while the segmentation results completed by MDCN and MGMLN ranged from 65.1% to 95.1%. The optimal weight of losses, with equal Γ_1_ and Γ_2__,_ was considered to achieve a relatively good performance for both segmentation and classification.

Though the proposed method achieved high performance in classification and segmentation, it presents a few limitations. Firstly, limited by the complexity and time cost of data collection and annotation, the dataset contained only two types of non-small cell carcinoma; therefore, the model cannot be widely applied in practical clinical diagnosis. In the future, we will expand the classification task to identify more types of lung cancer (e.g., large-cell carcinoma, small-cell lung cancer). Secondly, due to the shortage of computing resources, original images were cropped into 480 × 480-pixel patches in the training process. We plan to upgrade our equipment, so that the neural network can be implemented to study and analyze original images directly. Thirdly, classification and segmentation are the main functions of CAD system. Future research will address topics such as the flow design of the system, the achievement of a unified public sample library and seamless and efficient clinical applications. We will concentrate on the development of computational pathology software for research and clinical use in order to allow pathologists to focus on higher-level decisions, such as the design of antineoplastic protocols integrating information of microscopic anatomy and clinical medicine.

## Figures and Tables

**Table 1:** Demographic and clinical information (mean ± standard deviation) of the studied lung cancer subjects.

Diagnosis	Age	Gender (M/F) ^1^	Cancer Staging (I/II/III) ^2^	Tumor Volume (cm^2^)
SCC	64.4 ± 7.8	14/1	10/4/1	10.74 ± 9.5
AD	53.8 ± 12.6	5/6	9/2/0	2.91 ± 2.1
NC	61.1 ± 9.1	8/2	-	-

^1^ M/F: male or female. ^2^ Cancer Staging: clinical Tumor Node Metastasis stage.

**Table 2:** Performance of classification and segmentation after assigning different weights to their respective losses.

Performance in Different Γ	Classification Performance (%)				Segmentation Performance (%)				
		ACC	SEN	SPE		DSC	SEN	PRE	IoU
Γ_1_:Γ_2_ = 0:1	SCC vs. NC	97.8	95.2	100	Mean	—	—	—	—
					SCC	—	—	—	—
	AD vs. NC	90.2	86.1	100	AD	—	—	—	—
					NC	—	—	—	—
Γ_1_:Γ_2_ = 0.5:1	SCC vs. NC	92.7	90.5	95	Mean	79.4	84.5	78.7	77.9
					SCC	94.0	91.6	96.8	88.9
	AD vs. NC	98.1	100	100	AD	68.7	63.3	75.4	52.4
					NC	93.8	96.6	91.1	88.2
Γ_1_:Γ_2_ = 1:1	SCC vs. NC	95.1	95	95.2	Mean	92.3	92.2	91.9	85.2
					SCC	93.5	90.1	97.3	87.9
	AD vs. NC	100	100	100	AD	89.0	89.4	88.6	80.2
					NC	94.5	97.2	89.7	87.5
Γ_1_:Γ_2_ = 1:0.5	SCC vs. NC	92.7	94.7	90.9	Mean	84.4	80.5	88.0	73.0
					SCC	89.3	82.7	97.2	80.8
	AD vs. NC	95.7	100	90.9	AD	73.6	61.4	83.5	56.6
					NC	90.3	97.3	83.3	81.5
Γ_1_:Γ_2_ = 1:0	SCC vs. NC	—	—	—	Mean	89.4	88.6	90.5	82.1
					SCC	95.6	94.2	97.8	92.3
	AD vs. NC	—	—	—	AD	76.8	73.9	80.1	62.4
					NC	95.9	97.6	93.7	91.7

Mean: average of the evaluation indexes of SCC, AD and NC.

**Table 3:** Comparison of our method with other multi-task methods for segmentation and classification.

Method	Tumor Type	Segmentation Performance (%)				Tumor Type	Classification Performance (%)		
		DSC	SEN	Pre	IoU		ACC	SEN	SPE
MGMLN	Mean	84.1	79.6	91.5	74.6	SCC vs. NC	64.1	100.0	30.0
	SCC	92.0	88.1	96.4	85.3				
	AD	65.1	52.2	86.9	48.4	AD vs. NC	96.6	100.0	85.7
	NC	95.1	98.5	91.3	90.0				
MDCN	Mean	81.4	81.7	85.3	71.1	SCC vs. NC	92.7	90.0	95.2
	SCC	85.2	74.6	99.6	74.4				
	AD	69.4	80.9	60.2	52.7	AD vs. NC	100.0	100.0	100.0
	NC	89.7	89.5	96.0	86.3				
MCN	Mean	92.3	92.2	91.9	85.2	SCC vs. NC	95.1	95.0	95.2
	SCC	93.5	90.1	97.3	87.9				
	AD	89.0	89.4	88.6	80.2	AD vs. NC	100.0	100.0	100.0
	NC	94.5	97.2	89.7	87.5				

## Data Availability

Not applicable.
